# Guidance for the prevention and treatment of cancer-associated venous thromboembolism

**DOI:** 10.1007/s11239-015-1313-4

**Published:** 2016-01-16

**Authors:** Alok A. Khorana, Marc Carrier, David A. Garcia, Agnes Y. Y. Lee

**Affiliations:** Taussig Cancer Institute and Case Comprehensive Cancer Center, Cleveland Clinic, 9500 Euclid Ave, R35, Cleveland, OH 44195 USA; Department of Medicine, The Ottawa Hospital Research Institute, University of Ottawa, Ottawa, ON Canada; Division of Hematology, University of Washington School of Medicine, Seattle, WA USA; Department of Medicine, Thrombosis Program, University of British Columbia and Vancouver Coastal Health Authority, Vancouver, BC Canada

**Keywords:** Venous thromboembolism, Prevention, Risk factors, Cancer, Direct oral anticoagulants (DOAC), New oral anticoagulants (NOAC)

## Abstract

Venous thromboembolism (VTE) is a highly prevalent complication of malignancy with emerging changes in incidence, diagnosis and treatment paradigms. This manuscript, initiated by the Anticoagulation Forum, provides clinical guidance based on existing guidelines and consensus expert opinion where guidelines are lacking. We address a) the appropriate workup to search for occult malignancy in patients with idiopathic VTE, b) identification of high-risk cancer patients for primary thromboprophylaxis, c) the appropriate immediate and long-term treatment for people with cancer diagnosed with acute thromboembolism, d) the appropriate duration of anticoagulation and e) the appropriate treatment strategy in patients with recurrent VTE on anticoagulation. Areas of controversy and future directions in this field are highlighted.

## Introduction

Thromboembolism frequently complicates the course of malignancy, particularly in the setting of medical and surgical anti-cancer treatments [1]. It can involve both the venous and arterial systems. The more common events are venous thromboembolism (VTE), including deep venous thrombosis (DVT) and pulmonary embolism (PE). VTE generally presents after the diagnosis of cancer but it can be the presenting symptom that leads to diagnosis of malignancy. Incidentally diagnosed VTE, particularly involving visceral or splanchnic veins such as the portal or superior mesenteric vein, are increasingly contributing to the burden of cancer-associated thrombosis [2]. Arterial events, such as stroke or myocardial infarction, are also much more prevalent in patients with malignancy, as compared to the general population.

Thrombosis in cancer patients is associated with deleterious consequences. Most important is the strong association with short- and long-term mortality; indeed, thromboembolism is the second-leading cause of death in patients with malignancy [3, 4]. VTE is also associated with recurrent VTE as well as bleeding, both at significantly higher rates than seen in non-cancer patients [3]. Finally, VTE is associated with a threefold increase in hospitalizations and higher total health care costs [5]. Thus, appropriate prevention and treatment of cancer-associated thrombosis is vital to reduce its burden on patients with malignancy and on the health system at large. This chapter will provide guidance on important clinical questions regarding risk assessment, primary prevention and treatment of cancer-associated VTE.

### Current guidelines and controversies

There has been a recent explosion of information regarding cancer-associated thrombosis; a PubMed keyword search identifies over 4600 published papers in just the past 5 years. Despite this wealth of knowledge, there remain several areas of controversy and incomplete knowledge. Although it is well recognized that VTE can be a presentation of occult malignancy, the breadth and depth of work-up to be initiated to search for occult cancer in a patient with unprovoked VTE is unclear. Second, despite the high overall prevalence of VTE in malignancy, there is wide variation amongst specific subgroups of cancer patients. Therefore, although primary prevention has been shown to be successful in various randomized trials, prophylaxis strategies need to be targeted toward those patients at highest risk. Selection of such patients is an important and controversial issue. Although current guidelines by the American Society of Clinical Oncology (ASCO), the European Society of Medical Oncology (ESMO) and the National Comprehensive Cancer Network (NCCN), are united in considering outpatient primary prophylaxis, patient selection strategies are not well-defined [6–8]. Next, the patient-friendly direct oral anticoagulants (DOACs) may offer an attractive alternative to other therapies; however, data in cancer patients and direct comparisons to the current standard of care with low molecular weight heparin (LMWH) are limited. Finally, little is known about the optimal duration of VTE treatment in malignancy or how to treat patients who experience recurrent thrombosis despite LMWH.

## Methods

To provide guidance on the prevention and treatment of cancer-associated thrombosis, we first developed a number of pivotal practical questions pertaining to the topic (Table 1). Questions were developed by consensus from the authors. The literature addressing the above questions was reviewed by searching electronic databases (PubMed, Medline), with a focus on high quality cohort studies and randomized controlled trials published in the last 10 years, where available, as well as on recent systematic reviews. For each question, a brief summary and interpretation of pertinent literature and existing guidelines, where available, are provided, followed by guidance to the reader.

**Table 1:** Guidance questions to be considered

1. What is the appropriate workup to search for occult malignancy in patients with idiopathic VTE?
2. How can high-risk cancer patients be identified for primary thromboprophylaxis?
3. What is the appropriate immediate and long-term treatment for people with cancer diagnosed with acute thromboembolism, including the role of DOACs?
4. What is the appropriate duration of anticoagulation?
5. What is the appropriate treatment strategy in patients with recurrent VTE on anticoagulation?

## Guidance

1. What is the appropriate workup to search for occult malignancy in patients with idiopathic VTE?

Patients with acute unprovoked VTE have a four-fold increased risk of having an underlying occult cancer compared to patients with provoking (e.g. recent surgery) risk factors [9]. Up to 10 % of patients with unprovoked VTE may be diagnosed with cancer within the first year following their thrombotic event [9]. The greatest period of detection is within the first 6 months [9–11] and the most frequently observed tumor types are cancers of the ovary, pancreas and liver [11]. The long term cumulative incidence of cancer diagnosis (i.e. beyond 6–12 months) has been reported to be comparable to the incidence described in the general population [12]. These data notwithstanding, the utility and extent of occult cancer screening is controversial. There is presently no consensus and wide variation in clinical practice regarding whether to perform occult cancer screening and what investigations should be included. The most recent version of the American College of Chest Physicians (ACCP) clinical practice guidelines does not provide a specific recommendation of occult cancer screening in patients with unprovoked VTE.

Whereas some studies have suggested that a limited occult cancer screening strategy, including medical history taking, physical examination, routine blood tests and a chest-X ray, is adequate to detect most occult cancers in patients with unprovoked VTE, others have shown that a more extensive occult cancer screening strategy (e.g. ultrasonography and/or computed tomography (CT) of the abdomen/pelvis, tumor markers, etc.) can increase the rate of detection and improve the sensitivity of screening. Only two studies have directly compared limited and extensive screening [13, 14]. A recent prospective cohort study comparing a limited occult cancer screening strategy to a strategy also including a mammography in women and thoracic and abdominal CT did not show any difference in the number of cancers subsequently diagnosed (5.0 vs. 3.7 %, respectively) or in overall mortality (8.3 vs. 7.6 %, respectively) during 2.5 years of follow up [13]. The only reported randomized controlled trial included patients with negative limited occult cancer screening and randomized them to no further testing or additional testing [14]. The extensive screening strategy had a sensitivity of 93 % and increased detection of the number of cases of early-stage cancers (T_1–2_, N_0_) (64 vs. 20 %, p = 0.047). An absolute risk reduction of cancer-related mortality of 1.9 % in favor of the extensive screening group during the 2-year follow-up period was reported. Although the lack of statistical significance of the cancer-related mortality difference might be due to lack of power, methodological limitations and implied possible lead-time bias undermine the results. Furthermore, the components of an ideal extensive occult cancer screening program are still unknown. A decision analysis using the data from the randomized trial described above and a meta-analysis have reported that limited occult cancer screening in combination with a CT abdomen/pelvis had the best yield [9, 15]. However, the complication rates, cost-effectiveness and difference in morbidity and mortality associated with this extensive screening could not be determined [9].

The National Institute for Health Care Excellence (NICE) guidelines on managing VTE suggest that all patients diagnosed with unprovoked VTE should be given a physical examination, chest X-ray, basic laboratory investigations, and urinanalysis. A CT abdomen/pelvis and, mammography for women, is also suggested in patients aged over 40 [16]. However, these recommendations are controversial as it remains unclear whether extensive occult cancer screening and earlier cancer detection improves morbidity, mortality and quality of life in patients with newly diagnosed VTE. Finally, extensive screening carries a significant economic cost and can induce significant psychological burden. Further clinical trials are required to assess the risks and benefits of an extensive occult cancer screening program in patients with unprovoked VTE.

### **Guidance Statements**

- *Patients with unprovoked VTE should undergo a through medical history and physical examination, basic laboratory investigations (complete blood counts, metabolic profile and liver function tests) and chest X-ray.*
- *We suggest that if not up-to-date, patients undergo age- and gender-specific cancer screening (i.e. cervical, breast, prostate and colon).*

(2) How can high-risk cancer patients be identified for primary thromboprophylaxis?

Although it is commonly stated that cancer patients are at high risk for VTE, there is significant variation in risk amongst subgroups of this population. In a large recent systematic review of studies, the VTE estimate for the general cancer population was approximately 13 per 1000 person-years (95 % CI 7–23) [17]. In patients with metastatic disease or those receiving thrombogenic regimens the risk was 68 per 1000 person-years (95 % CI 48–96) and as high as 200 per 1000 person-years (95 % CI 162–247) amongst patients with primary brain tumors. Discriminating between low- and high-risk patients is therefore crucial to optimize the risk-benefit ratio of thromboprophylaxis.

Clinical risk factors, biomarkers or combinations of the two can be used to estimate VTE risk. *Clinical risk factors* include the primary site of cancer (with highest rates observed in patients with primary brain tumors and cancers of the pancreas, stomach, liver, lungs and kidneys and hematologic malignancies including lymphomas and myeloma), advanced stage and therapeutic interventions including surgery, type of chemotherapy, erythropoiesis-stimulating agents, and devices such as central venous catheters and inferior vena cava filters (reviewed in [1]). It should be noted that the advent of novel “targeted” anti-cancer therapies to supplement or replace traditional chemotherapy-based regimens has not reduced the risk of VTE. Indeed, drugs targeting angiogenesis such as bevacizumab, sunitinib, sorafenib, the multi-targeted tyrosine kinase inhibitor ponatinib and the immunomodulator lenalidomide have been associated with arterial thromboembolism [18, 19], immunomodulatory agents such as thalidomide and lenalidomide have been associated with very high rates of VTE [20], and anti-epidermal growth factor antibodies such as cetuximab and panitumumab have also recently been associated with VTE [21]. A variety of candidate *biomarkers* have also been associated with VTE in malignancy. These include elevated platelet and leukocyte counts, decreased hemoglobin, elevated D-dimer, elevated prothrombin activation products, elevated soluble P-selectin, thrombin generation and elevated levels of TF-bearing microparticles (TFMP) [22].

Despite the multitude of reports linking cancer-associated VTE to individual risk factors or biomarkers, it should be noted that many of the published studies are univariate or limited multivariate analyses. Strategies utilizing such individual factors to enroll patients onto clinical trials of thromboprophylaxis have not yielded the event rates that would have been predicted. For instance, in two large trials of outpatient prophylaxis, patients were selected based only on primary site and advanced stage; in these studies, event rates in the placebo arm ranged from 3.4 to 3.9 % and even though the studies found a statistically significant reduction with thromboprophylaxis in symptomatic VTE, results were not adopted by the oncology community due to low event rates [23, 24]. Based on this experience, the latest ASCO guidelines recommend against the use of single risk factors to identify high-risk patients [7].

The ASCO panel and other guidelines including NCCN and ESMO instead recommend the use of a validated *risk assessment tool* to discriminate between high- and low-risk patients (Table 2). This risk score was originally derived from a development cohort of 2701 patients and then validated in an independent cohort of 1365 patients from a prospective registry by Khorana and colleagues (the so-called “Khorana Score”) [25]. Subsequently, the Score was externally validated prospectively by the Vienna CATS consortium and multiple retrospective cohort studies [1, 26].

**Table 2:** Predictive model for VTE according to Khorana et al. [25]

Patient characteristics	Risk score
Site of cancer	
Very high risk (stomach, pancreas)	2
High risk (lung, lymphoma, gynecologic, bladder, testicular)	1
Prechemotherapy platelet count ≥350,000/mm^3^	1
Hemoglobin level less than 10 g/dl or use of red cell growth factors	1
Prechemotherapy leukocyte count >11,000/mm^3^	1
Body mass index ≥35 kg/m^2^ or more	1

High-risk score ≥3

Intermediate risk score 1–2

Low-risk score 0

How can risk stratification be utilized to select patients for outpatient thromboprophylaxis? An updated Cochrane systematic review of multiple randomized trials of outpatient prophylaxis in malignancy found that LMWH significantly reduced symptomatic VTE (RR 0.62, 95 % CI 0.41–0.93) but with a relatively high number needed to treat (NNT = 60) [27]. LMWH was associated with a 60 % non-significant increase in major bleeding (RR 1.57, 95 % CI 0.69–3.60). Thus patients with higher absolute risk of VTE would derive greater benefit and conversely patients with a lower baseline risk would derive less benefit or no benefit. Proof of this concept comes from subgroup analyses of the two largest randomized trials. Rates of VTE in high-risk patients (Khorana Score ≥3) enrolled in PROTECHT were 11.1 % in the placebo arm and 4.5 % in the nadroparin arm (NNT = 15, compared to 77 for low- and intermediate-risk patients) [28]. Similarly, in a per-protocol subgroup analysis of SAVE-ONCO, NNT was 25 for high-risk patients compared to 333 for low-risk patients [29]. No differences were observed in bleeding rates between high- and low-risk patients. Based on these findings, prophylaxis is not recommended in unselected general cancer patients (i.e. without risk stratification) or in those with a high risk of bleeding (e.g. primary brain tumors).

One niche population in this regard is patients with multiple myeloma receiving imid-based regimens. In an updated Cochrane meta-analysis, LMWH was associated with a significant reduction in symptomatic VTE when compared with the vitamin K antagonist warfarin (RR 0.33, 95 % CI 0.14–0.83), while the difference between LMWH and aspirin was not statistically significant (RR 0.51, 95 % CI 0.22–1.17) [27].

It should be noted that there are other settings in which pharmacologic thromboprophylaxis is generally recommended by major guidelines panels. These include hospitalized cancer patients with other risk factors such as an acute medical illness or recent surgery. In acutely ill medical inpatients, unfortunately, no cancer-specific clinical trials have been conducted; recommendations are therefore made based upon extrapolation of data from randomized trials that included only a small minority of cancer patients [30]. Extended prophylaxis with LMWH for up to 4 weeks postoperatively should be considered for patients undergoing major abdominal or pelvic surgery for cancer who have high-risk features such as restricted mobility, obesity or history of VTE [7]. There are currently no substantial data on the use of DOACs in the prophylaxis setting; ongoing studies are addressing this issue.

### **Guidance Statements**

*(See Table* 3*for dosing): Recommendations are made assuming no existing contraindications to pharmacologic prophylaxis.*

**Table 3:** Dosing regimens for prevention and treatment of VTE in patients with malignancy (Adapted from ASCO [7])

Drug	Regimen
Pharmacologic (anticoagulant) prophylaxis^a^	
Hospitalized medical patients^b^	
Unfractionated heparin	5000 U once every 8 h^c^
Dalteparin	5000 U once daily
Enoxaparin	40 mg once daily
Fondaparinux	2.5 mg once daily
Surgical patients^b,d^	
Unfractionated heparin	5000 U 2–4 h preoperatively and once every 8 hours thereafter or 5000 U 10–12 h preoperatively and 5000 U once daily thereafter^c^
Dalteparin	2500 U 2–4 h preoperatively and 5000 U once daily thereafter or 5000 U 10–12 h preoperatively and 5000 U once daily thereafter
Enoxaparin	20 mg 2–4 h preoperatively and 40 mg once daily thereafter or 40 mg 10–12 h preoperatively and 40 mg once daily thereafter
Fondaparinux	2.5 mg qd beginning 6–8 h postoperatively
Treatment of established VTE	
Initial	
Unfractionated heparin^e^	80 U/kg IV bolus, then 18 U/kg per hour IV; adjust dose based on aPTT^h^
Dalteparin^e,g,h^	100 U/kg once every 12 h; 200 U/kg once daily
Enoxoparin^e,g,h,i^	1 mg/kg once every 12 h; 1.5 mg/kg once daily
Tinzaparin^e,g,h,j^	175 U/kg once per day
Fondaparinux^e,g^	<50 kg, 5.0 mg once daily; 50–100 kg, 7.5 mg once daily; >100 kg, 10 mg once daily
Long term^k^	
Dalteparin^h,g^	200 U/kg once daily for 1 month, then 150 U/kg once daily
Enoxaparin^g,h,i^	1.5 mg/kg once daily; 1 mg/kg once every 12 h
Tinzaparin^h,j^	175 U/kg once daily
Warfarin	Adjust dose to maintain INR 2–3

*aPTT* activated partial thromboplastin time, *FDA* US Food and Drug Administration, *INR* international normalized ratio, *IV* intravenous, *LMWH* low-molecular weight heparin, *VTE* venous thromboembolism

^a^All doses are administered as subcutaneous injections except as indicated

^b^Duration for medical patients is length of hospital stay or until fully ambulatory; for surgical patients, prophylaxis should be continued for at least 7–10 days. Extended prophylaxis for up to 4 weeks should be considered for high-risk patients

^c^Unfractionated heparin 5000 U every 12 h has also been used but appears to be less effective

^d^When neuraxial anesthesia or analgesia is planned, prophylactic doses of once-daily LMWH should not be administered within 10–12 h before the procedure/instrumentation (including epidural catheter removal). After surgery, the first dose of LMWH can be administered 6–8 h postoperatively. After catheter removal the first dose of LMWH can be administered no earlier than 2 h afterward. Clinicians should refer to their institutional guidelines and the American Society of Regional Anesthesia Guidelines for more information

^e^Parenteral anticoagulants should overlap with warfarin for 5–7 days minimum and continued until INR is in the therapeutic range for 2 consecutive days

^f^Unfractionated heparin infusion rate should be adjusted to maintain the aPTT within the therapeutic range in accordance with local protocol to correspond with a heparin level of 0.3–0.7 U/mL using a chromogenic Xa essay

^g^Dependent on significant renal clearance; avoid in patients with creatinine clearance ≤30 mL/min or adjust dose based on anti-factor Xa levels

^h^Optimal dose unclear in patients >120 kg

^i^Twice-daily dosing may be more efficacious than once-daily dosing for enoxaparin based on post hoc data

^j^This drug is not available in the United States

^k^Total duration of therapy depends on clinical circumstances. See Clinical Question 4, section entitled “Initial Long-Term Treatment Up to 6 Months,” for more detailed discussion

- *We suggest against routine thromboprophylaxis in unselected and low-risk cancer outpatients. We also suggest against routine thromboprophylaxis in patients with high risk for bleeding (e.g. primary brain tumors).*
- *We suggest consideration of outpatient thromboprophylaxis with LMWH in high-risk (Khorana Score ≥3 or advanced pancreas) cancer outpatients receiving chemotherapy and with aspirin or LMWH in patients with myeloma receiving imid-based regimens.*
- *We suggest routine consideration of inpatient thromboprophylaxis with LMWH or unfractionated heparin in cancer patients hospitalized with an acute medical illness.*
- *We suggest inpatient thromboprophylaxis with LMWH or unfractionated heparin in cancer patients undergoing major surgery.*
- *We suggest post-operative thromboprophylaxis with LMWH for up to 4 weeks in patients undergoing major abdominal or pelvic surgery for cancer with high-risk features such as immobility, obesity and history of VTE.*

(3) What is the appropriate immediate and long-term treatment for people with cancer diagnosed with acute VTE, including the role of DOACs?

Cancer patients with VTE have higher rates of complications, including a 12 % annual risk of bleeding complications and up to a 21 % annual risk of recurrent VTE while on warfarin therapy [3]. Furthermore, epidemiologic and other studies have suggested that cancer-associated VTE may be relatively resistant to warfarin. Therefore LMWHs were evaluated as an alternate, extended-therapy option to warfarin. The CLOT trial reported by Lee et al. randomized 676 cancer patients with VTE to receive initial dalteparin followed by 6 months of either dalteparin or warfarin with target INR 2.5 [31]. Fifteen percent of patients treated with warfarin developed recurrent VTE compared to 7.9 % of patients treated with dalteparin [hazard ratio (HR) 0.48, 95 % CI 0.30–0.77; NNT = 12). This study established the superiority of LMWH for long-term anticoagulation in cancer patients. This and subsequent smaller studies have been evaluated in a Cochrane systematic review that further supports the initial findings of the CLOT study [32]. A recent presentation of the CATCH trial, the largest treatment study of cancer-associated thrombosis, largely confirms these initial findings [33, 34]. In this global, randomized phase III clinical trial, 900 patients were randomized to either tinzaparin 175 IU/kg once daily for 6 months or initial tinzaparin 175 IU/kg once daily for 5–10 days overlapped and followed by dose-adjusted warfarin (target INR 2–3) for 6 months. Over the 6-month trial period, 31 patients (6.9 %) in the tinzaparin arm experienced recurrent VTE compared with 45 (10 %) in the warfarin arm [HR 0.65 (95 % CI 0.41–1.03; p = 0.07)]. Symptomatic non-fatal DVT occurred in 12 patients (2.7 %) in the tinzaparin arm and 24 (5.3 %) in the warfarin arm [HR 0.48 (95 % CI 0.24–0.96); p = 0.04]. Significantly fewer patients experienced clinically relevant non-major bleeding with tinzaparin than warfarin (11 vs. 16 %, respectively; p = 0.03). There were no differences in rates of major bleeding, non-fatal PE or mortality between the two arms. Current guidelines recommend long-term anticoagulation with LMWH for cancer patients with VTE as the preferred approach and results of this latest trial support this strategy [7].

**Fig. 1:**
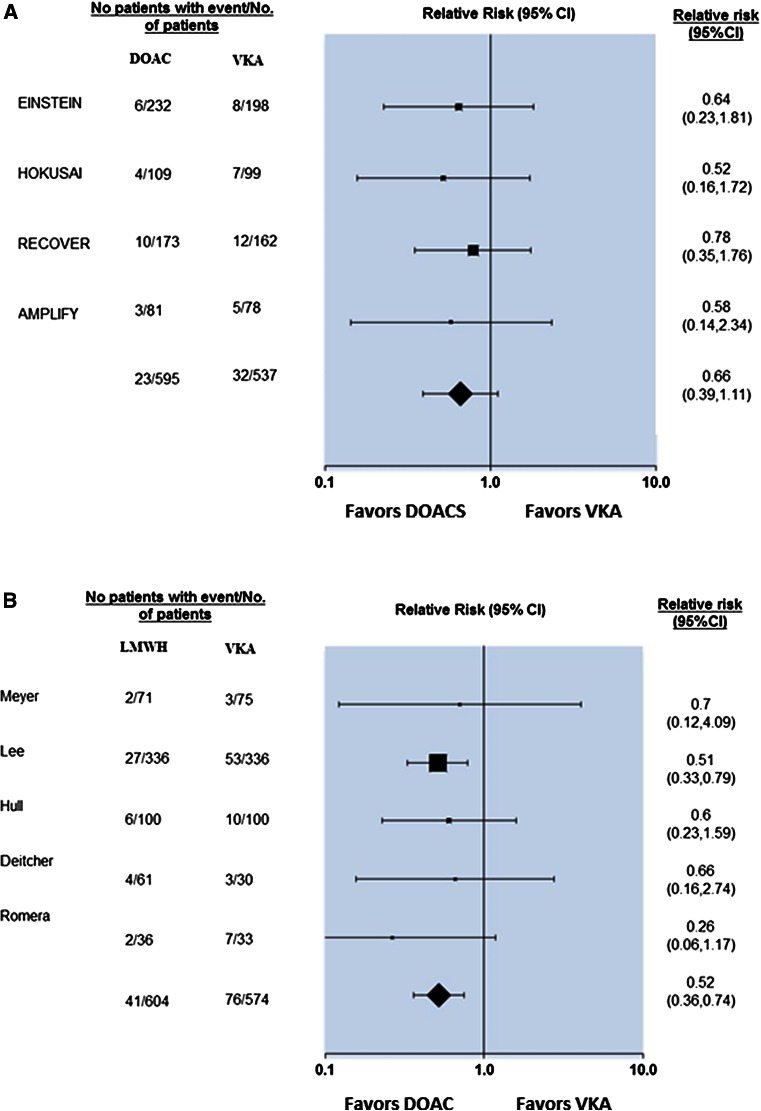
Forest plot of relative risks across clinical trials comparing (**a**) DOAC versus VKA and (**b**) LMWH alone versus VKA for recurrent cancer-associated VTE. The definition of active cancer and therefore of the study population varied across trials

The past few years have seen the emergence of several DOACs, which have been shown to be comparable to conventional therapy with warfarin for the acute treatment of VTE. Four agents (rivaroxaban, dabigatran, apixaban and edoxaban) have received regulatory approval for the treatment of VTE. However, none of these agents were tested in cancer-specific populations and, in all of the treatment studies, patients in the control arm received vitamin K antagonists (VKA) rather than LMWH. The definition of “active cancer” was also not consistent across studies, and some included cancer survivors. Therefore, the efficacy and safety of DOACs in patients with cancer-associated VTE remains uncertain. Indeed, the European Medecines Agency label for apxiaban notes that its efficacy and safety in patients with active cancer has not been established [35]. A recent meta-analysis evaluated 9 randomized trials involving 2310 patients with cancer-associated thrombosis treated with DOACs [36]. In comparison to VKA, LMWH showed a significant reduction in recurrent VTE events (RR: 0.52; 95 % CI 0.36–0.74) whereas DOACs did not (RR: 0.66; 95 % CI 0.39–1.11) (Fig. 1). LMWH was associated with a non-significant increase in the risk of major bleeding (RR: 1.06; 95 % CI 0.5–2.23) whereas DOACs showed a non-significant reduction (RR: 0.78; 95 % CI 0.42–1.44) compared to VKA. Annualized risks of recurrent VTE and major bleeding among patients randomized to VKA were higher in the LMWH studies as compared to the studies assessing DOACs, suggesting that a higher risk cancer population was enrolled in the LMWH studies. Ongoing and planned studies aim to determine the relative safety and efficacy of DOACs in cancer-associated VTE compared with LMWH.

Incidental VTE, defined as VTE discovered on scans ordered for reasons other than suspected VTE (typically cancer staging or restaging) is an emerging major contributor to the burden of cancer-associated VTE. Although management of these events remains controversial, retrospective studies have found similar risks of mortality and recurrent VTE between patients with symptomatic and incidental PE [37–40]. Given the high risk of future, symptomatic VTE in these patients, many clinicians are reluctant to manage them without anticoagulation. However, there is some evidence that patients with isolated, incidental subsegmental PE may not need anticoagulant treatment [41].

Further confusion regarding incidental VTE surrounds the diagnosis of incidental visceral vein thrombi. Indeed, the majority of incidental VTE in malignancy involves visceral veins [38]. Visceral vein thrombi include portal, mesenteric, splenic, renal and gonadal vein thrombi. In a cohort study of gastrointestinal cancer patients, 100 % of visceral vein thrombi were incidentally discovered compared to 35 % of PE [42]. The consequences of incidental visceral vein thrombi are less well understood, although there appears to be at least an independent association with mortality [(HR) 2.6, 95 % CI 1.6–4.2] in patients with pancreas cancer [38]. It is unclear whether this association with mortality can be ameliorated by anticoagulation. Due to lack of evidence, decisions to treat or not treat visceral vein thrombi are made inconsistently, largely based on provider opinion and anecdotal experience. In a study by Ageno and colleagues, one-half of abdominal vein thrombi were not treated with anticoagulation [43]. Prospective clinical trials data in this setting are sorely lacking.

### **Guidance Statements**

*(see Table* 3*for dosing):*

- *We suggest that patients with active cancer (i.e. known disease or receiving some form of anti-cancer therapy) and VTE be treated with LMWH for at least 6 months.*
- *We suggest that patients with incidentally diagnosed DVT or PE be treated similarly to patients diagnosed with VTE based on symptoms i.e., with at least 6 months of LMWH monotherapy, with the exception of isolated subsegmental PE where decisions can be made on a case-by-case basis. We further suggest that treatment decisions in patients with incidentally diagnosed visceral vein thrombi be made on a case-by-case basis.*

(4) What is the appropriate duration and preferred agent for anticoagulation in cancer patients with VTE?

The optimal duration of anticoagulation is not known as this has not been formally assessed beyond 6 months. In current practice, the consensus is to continue anticoagulation for at least 6 months and then reassess the need for continuing anticoagulation. In those with ongoing risk factors, such as metastatic or progressive disease or ongoing systemic chemotherapy, continuing anticoagulation may be indicated to prevent recurrence. Conversely, in those without ongoing risk factors, the risk of recurrent VTE is likely sufficiently low to justify stopping anticoagulation.

Anticoagulation may be continued beyond 6 months if there is active malignancy and/or active, ongoing anti-neoplastic therapy. Results from a post-marketing study of extended treatment with dalteparin in patients with malignancy (DALTECAN) were recently reported. Of 334 patients, 109 (33 %) completed 12 months of dalteparin. Median treatment duration was 214 days. The highest major bleeding rate was in the first month of dalteparin therapy at 3.6 %, declining to 1.1 % during months 2–6, and 0.7 % over months 7–12 (p = 0.39 for months 2–6 vs. 7–12). The incidence of new or recurrent VTE was 11.1 %; again, highest for month 1 at 5.7 %, falling to 0.8 % per month for months 2–6 and 0.7 % per month for months 7–12. These data suggest that extended treatment is feasible and major bleeding is not a substantial concern; however, risk-benefit ratio is unclear given relatively low rates of recurrent VTE past initial months of treatment. Given lack of randomized trial evidence, the best agent in this setting is unknown.

### **Guidance Statements**

- *Anticoagulation with LMWH monotherapy should be prescribed for a minimum period of 6 months after diagnosis of cancer-associated VTE. Anticoagulation therapy should be continued beyond 6 months if a patient has active malignancy (i.e. persistent malignant disease) or if ongoing anti-cancer therapy is planned.*
- *For patients at low risk of recurrence we suggest that anticoagulation be discontinued after 6 months in the absence of active malignancy (i.e. patients are cured or in complete remission), provided that no anti-cancer therapy is ongoing or planned.*
- *For patients at high risk of recurrence we suggest that anticoagulation be continued but with periodic re-evaluation of risks and benefits.*

(5) What is the appropriate treatment strategy in patients with recurrent VTE on anticoagulation?

As noted earlier, recurrent VTE is not infrequent even in patients receiving appropriate anticoagulation in the setting of malignancy. Unfortunately, there are no randomized trials to provide an evidence-based approach. A recent paper and an ISTH guidance statement have described empiric approaches to this clinical problem [44, 45]. In general, LMWH monotherapy is considered the preferred approach. If patients are already on LMWH, dose escalation should be considered [44]. In a retrospective cohort study, the weight-adjusted dose of LMWH was increased by 20–25 % for at least 4 weeks. Patients on maintenance dose of LMWH were increased to full therapeutic dose for 6–12 weeks. Only 8.6 % of patients had a second recurrent VTE with this approach and 4.3 % had bleeding complications.

Inferior vena cava (IVC) filters should only be used temporarily in patients with acute thrombosis who have absolute contraindications to anticoagulation. In a prospective randomized study of 200 patients (including 56 with cancer), patients who received filters had short-term protection from PE but higher rates of DVT and filter-site thrombosis compared to those randomized to no filters (20.8 vs. 11.6 %, OR 1.87, 95 % CI 1.10–1.38). No short- or long-term survival benefit from IVC filter placement was seen. The potential risks of IVC filter placement are highlighted by non-randomized cohort studies that found IVC filters were associated with increased metastases and reduced survival in cancer patients [46].

### **Guidance Statements**

- *We suggest that cancer patients with symptomatic recurrent VTE despite therapeutic anticoagulation with an agent other than LMWH be transitioned to therapeutic LMWH, assuming no contraindications to LMWH.*
- *We suggest that cancer patients with symptomatic recurrent VTE despite optimal anticoagulation with LMWH continue with LMWH at a higher dose, starting at an increase of ~25 % of the current dose or resuming the therapeutic weight-adjusted dose if the patient was receiving a non-therapeutic dose at the time of recurrence.*
- *We suggest against the use of IVC filters except in the presence of absolute contraindications to pharmacologic anticoagulation (e.g. active bleeding). If necessary, retrievable filters should be used and a plan created to retrieve the filter when appropriate.*

## Conclusion

VTE is an important cause of morbidity and mortality in patients with malignancy. In this chapter, we have identified important clinical questions and attempted to provide recommendations to clinicians based on analysis of existing data, systematic reviews and meta-analyses as well as consensus between authors (Table 4). Many important issues remain to be addressed; in particular, how best to enhance appropriate utilization of outpatient thromboprophylaxis and how to integrate the emerging class of DOACs into prevention and treatment of malignancy. Considering the intense scientific interest in this area that has emerged in the past decade, we are cautiously optimistic that the scientific community can continue to identify ways to enhance patient-centered care and reduce the public health burden of this important and consequential complication of malignancy and its treatments.

**Table 4:** Summary of guidance statements

Question	Guidance statement
1. What is the appropriate workup to search for occult malignancy in patients with idiopathic VTE?	Patients with unprovoked VTE should undergo a through medical history and physical examination, basic laboratory investigations (complete blood counts, metabolic profile and liver function tests) and chest X-ray We suggest that if not up-to-date, patients undergo age- and gender-specific cancer screening (i.e. cervical, breast, prostate and colon)
2. How can high-risk cancer patients be identified for primary thromboprophylaxis?	Recommendations are made assuming no existing contraindications to pharmacologic prophylaxis We suggest against routine thromboprophylaxis in unselected and low-risk cancer outpatients. We also suggest against routine thromboprophylaxis in patients with high risk for bleeding (e.g. primary brain tumors) We suggest consideration of outpatient thromboprophylaxis with LMWH in high-risk (Khorana Score ≥3 or advanced pancreas) cancer outpatients receiving chemotherapy and with aspirin or LMWH in patients with myeloma receiving imid-based regimens We suggest routine consideration of inpatient thromboprophylaxis with LMWH or unfractionated heparin in cancer patients hospitalized with an acute medical illness We suggest inpatient thromboprophylaxis with LMWH or unfractionated heparin in cancer patients undergoing major surgery We suggest post-operative thromboprophylaxis with LMWH for up to 4 weeks in patients undergoing major abdominal or pelvic surgery for cancer with high-risk features such as immobility, obesity and history of VTE
3. What is the appropriate immediate and long-term treatment for people with cancer diagnosed with acute thromboembolism, including the role of DOACs?	We suggest that patients with active cancer (i.e. known disease or receiving some form of anti-cancer therapy) and VTE be treated with LMWH for at least 6 months We suggest that patients with incidentally diagnosed DVT or PE be treated similarly to patients diagnosed with VTE based on symptoms i.e., with at least 6 months of LMWH monotherapy, with the exception of isolated subsegmental PE where decisions can be made on a case-by-case basis. We further suggest that treatment decisions in patients with incidentally diagnosed visceral vein thrombi be made on a case-by-case basis
4. What is the appropriate duration of anticoagulation?	Anticoagulation with LMWH monotherapy should be prescribed for a minimum period of 6 months after diagnosis of cancer-associated VTE. Anticoagulation therapy should be continued beyond 6 months if a patient has active malignancy (i.e. persistent malignant disease) or if ongoing anti-cancer therapy is planned For patients at low risk of recurrence we suggest that anticoagulation be discontinued after 6 months in the absence of active malignancy (i.e. patients are cured or in complete remission), provided that no anti-cancer therapy is ongoing or planned For patients at high risk of recurrence we suggest that anticoagulation be continued but with periodic re-evaluation of risks and benefits
5. What is the appropriate treatment strategy in patients with recurrent VTE on anticoagulation?	We suggest that cancer patients with symptomatic recurrent VTE despite therapeutic anticoagulation with an agent other than LMWH be transitioned to therapeutic LMWH, assuming no contraindications to LMWH We suggest that cancer patients with symptomatic recurrent VTE despite optimal anticoagulation with LMWH continue with LMWH at a higher dose, starting at an increase of ~25 % of the current dose or resuming the therapeutic weight-adjusted dose if the patient was receiving a non-therapeutic dose at the time of recurrence We suggest against the use of IVC filters except in the presence of absolute contraindications to pharmacologic anticoagulation (e.g. active bleeding). If necessary, retrievable filters should be used and a plan created to retrieve the filter when appropriate
